# 4260 Hollow, Degradable poly(N-Isopropylacrylamide) Derived Nanoparticles for the Delivery of Anti-Inflammatory Peptides for the Treatment and Prevention of Post-Traumatic Osteoarthritis

**DOI:** 10.1017/cts.2020.334

**Published:** 2020-07-29

**Authors:** Marcus A Deloney, Kyra Smart, Blaine Christiansen, Alyssa Panitch

**Affiliations:** 1 University of California, Davis

## Abstract

OBJECTIVES/GOALS: Knocking down the inflammatory response following joint trauma may halt the cytokine cascade and prevent the resulting cyclic degradation of articular cartilage. MK2 inhibiting (MK2i) peptides are an emerging and promising class of pharmaceutical to treat post-traumatic osteoarthritis (PTOA); however, these peptides are susceptible to proteolytic degradation in the extracellular space. Our objective is to encapsulate MK2i in thermoresponsive hollow nanoparticles (hNPs) to knockdown the inflammatory cytokine IL-6 to prevent the cyclic degradation of articular cartilage. METHODS/STUDY POPULATION: *NP Synthesis:* N-isopropyl acrylamide (NIPAm) cores was initiated by potassium persulfate (KPS) in aqueous solution with sodium dodecylsulfate (SDS) at 70°C under a nitrogen for 2 hours. Then exposed to oxygen for 45 min, followed by a nitrogen purge. NIPAm, 2-acrylamindo-2-methyl-1-propanesulfonic acid (AMPS), N,N’-bis(acryoyl)cystamine (BAC), and Acrylic Acid (AAc), in fluorescent batches rhodamine b isothiocyanate (RBITC), were polymerized around the core to form the shell. NPs were purified using tangential flow filtration. The NPs were dialyzed at 4°C for 14 days to remove the core and form hNPs. *Loading & Release:* hNPs and MK2i were incubated at 1 mg/ml at 4°C for 24 h. MK2i released into 1x PBS and analyzed on HPLC. *IL-6 Expression*: Bovine chondrocytes seeded at 10,000 cell/cm^2^ were stimulated with 20 ng/ml IL-1b daily and treated once with 100 µg/ml MK2i loaded-NP or 100 µg/ml free MK2i treatment on day 2. Analyzed on bovine IL-6 ELISA. *In Vivo Intra Articular Injections:* 75 µl of 2 mg/ml hNPsRHB or a PBS control was injected into the right knee of 4-month old Fischer 344 (Envigo) rats. Rats were imaged daily for 7 days then euthanized, legs dissected, and imaged. RESULTS/ANTICIPATED RESULTS: Core removal facilitated increased MK2i release from hNPs, Fig 1A, allowing up to 63% after 5 days in PBS. The hNPs generated here offer a continual sustained release of MK2i and hNPs are non-cytotoxic (data not shown) up to 12 mg/ml. MK2i loaded-NPs significantly knocked down IL-6 production after a single treatment after 2 days, Figure 1B, and continued knockdown for up to 4 days. hNPsRBITC was successfully injected into rat joint space and was retained for at least 7-days compared to pre-injection and PBS control, Fig 1 B-C. DISCUSSION/SIGNIFICANCE OF IMPACT: hNPs protect MK2i from ECM degradation and offer continual sustained release into chondrocytes. Core removal allows for MK2i release *in vitro* with further sustained release compared to previous non-degradable model. The single MK2i treatment lead to a significant IL-6 knockdown bovine chondrocytes for up to 4 days in hNPs. We were able to successfully inject and retain fluorescently labeled hNPs within rat knees for 7 days. Our translational therapeutic shows the promise of delivering a degradable, non-cytotoxic hNP into the joint space to knockdown the inflammatory response to halt the cyclic progression of articular cartilage degradation and progression of PTOA. CONFLICT OF INTEREST DESCRIPTION: The authors declare the following competing financial interest(s): Moerae Matrix, Inc. has a worldwide exclusive license to the CPP (MK2 inhibitor peptide). A. Panitch owns greater than 5% of Moerae Matrix, Inc.

